# Topical oxidized regenerated cellulose for perioperative blood management in total hip and knee arthroplasty: a systematic review and meta-analysis

**DOI:** 10.3389/fsurg.2026.1900156

**Published:** 2026-09-18

**Authors:** Wenbo Hou, Jie Wang, Jun Liu, Lei Wang

**Affiliations:** Department of Joint Surgery, Tianjin Hospital of Tianjin University, Tianjin, China

**Keywords:** meta-analysis, oxidized regenerated cellulose, perioperative blood loss, surgicel, topical hemostatic agent, total hip arthroplasty, total knee arthroplasty, tranexamic acid

## Abstract

**Background:**

Total hip arthroplasty (THA) and total knee arthroplasty (TKA) are established procedures for end-stage joint disease, but perioperative blood loss remains a practical concern. Oxidized regenerated cellulose (ORC) has been used as a local hemostatic material during arthroplasty; however, its comparative value in contemporary blood-management protocols remains uncertain.

**Methods:**

PubMed, Embase, the Cochrane Library, and Scopus were searched from inception to March 2026. Randomized controlled trials and comparative observational studies were eligible if they evaluated ORC in THA or TKA and reported blood-loss-related or complication-related outcomes. Continuous outcomes were pooled as mean differences (MDs), and dichotomous outcomes as risk ratios (RRs), with 95% confidence intervals (CIs). Random-effects models were used.

**Results:**

Eleven studies were included. Compared with no ORC or standard care, ORC was associated with lower total perioperative blood loss (MD = −124.77 mL, 95% CI −181.71 to −67.83; *P* < 0.0001), postoperative or hidden blood loss (MD = −136.54 mL, 95% CI −214.97 to −58.10; *P* = 0.0006), and hemoglobin drop/loss (MD = −0.57 g/dL, 95% CI −0.81 to −0.34; *P* < 0.00001). No significant differences were found for intraoperative blood loss, transfusion rate, thrombotic events, or overall complications. Compared with active topical comparators, ORC showed no clear advantage. Evidence for ORC plus TXA vs. TXA alone was based on a single study and should be interpreted as exploratory. These effects mainly represent surrogate outcomes rather than definitive patient-important benefits.

**Conclusions:**

ORC may modestly reduce blood-loss-related surrogate outcomes when compared with no ORC or standard care. Current evidence does not show clear superiority over active topical comparators or establish definite incremental benefit in TXA-based protocols. Because overall certainty remains limited, routine ORC use in unselected arthroplasty patients is not supported; future trials should focus on patient-important outcomes.

**Registration:**

PROSPERO registration number: CRD420261384870.

## Introduction

### Background

Total hip arthroplasty (THA) and total knee arthroplasty (TKA) are among the most successful and widely performed procedures for the treatment of end-stage joint diseases, including osteoarthritis and rheumatoid arthritis (1, 2). With population aging and increasing surgical demand, the number of primary and revision arthroplasties continues to rise worldwide (3). Despite advances in surgical techniques and perioperative care, substantial perioperative blood loss remains a common concern because it may contribute to postoperative anemia, delayed recovery, transfusion requirements, and increased healthcare costs (4).

Perioperative blood conservation in arthroplasty relies on several complementary strategies, including careful surgical technique, appropriate tourniquet use, and pharmacological interventions. Tranexamic acid (TXA) is now a central component of many blood-management protocols because it reduces blood loss and transfusion requirements without a clear increase in thromboembolic risk (5, 6). Even so, topical hemostatic approaches remain relevant, particularly when surgeons seek additional local control of diffuse oozing or when patient factors make a purely systemic approach less desirable (6).

Oxidized regenerated cellulose (ORC) is an absorbable topical hemostatic material that supports clot formation by providing a matrix for platelet adhesion and aggregation and by creating an acidic local environment (7, 8). In THA and TKA, this mechanism may be useful for broad or irregular bleeding surfaces, including cancellous bone, osteotomy sites, the acetabulum, femoral medullary canal, and periarticular soft tissues (9–19). Powdered or fibrillar formulations may be applied directly to deep or uneven surfaces where thermal coagulation or suturing is less practical (9–19).

Available evidence is mixed and context-dependent (9–19). Studies comparing ORC with no local hemostatic material generally reported reductions in blood-loss-related measures, whereas studies using active topical comparators such as topical TXA or gelatin-thrombin hemostatic matrix (GTHM) did not show clear superiority of ORC (9–19). Evidence on ORC as an adjunct to TXA is limited. Differences in procedure, formulation, application site, TXA use, tourniquet and drainage protocols, follow-up timing, and calculation methods further complicate interpretation.

Therefore, the key clinical question is whether ORC provides clinically meaningful additional value in contemporary arthroplasty blood-management protocols, particularly in the TXA era. Using the PICOS framework, we evaluated patients undergoing THA or TKA, intraoperative topical ORC, and three comparator settings: no ORC or standard control, active topical hemostatic comparators, and ORC as an adjunct to TXA. Outcomes included blood-loss-related measures, transfusion, thrombotic events, overall complications, and reported recovery-related outcomes.

## Methods

### Study design and reporting standards

This systematic review and meta-analysis was conducted in accordance with the Preferred Reporting Items for Systematic Reviews and Meta-Analyses (PRISMA) guidelines (20). The completed PRISMA flow diagram is presented in Figure 1, and the PRISMA checklist is provided as an additional file. The protocol was registered in PROSPERO (registration number: CRD420261384870). Because this study was based exclusively on previously published data, ethical approval and informed consent were not required.

**Figure 1:**
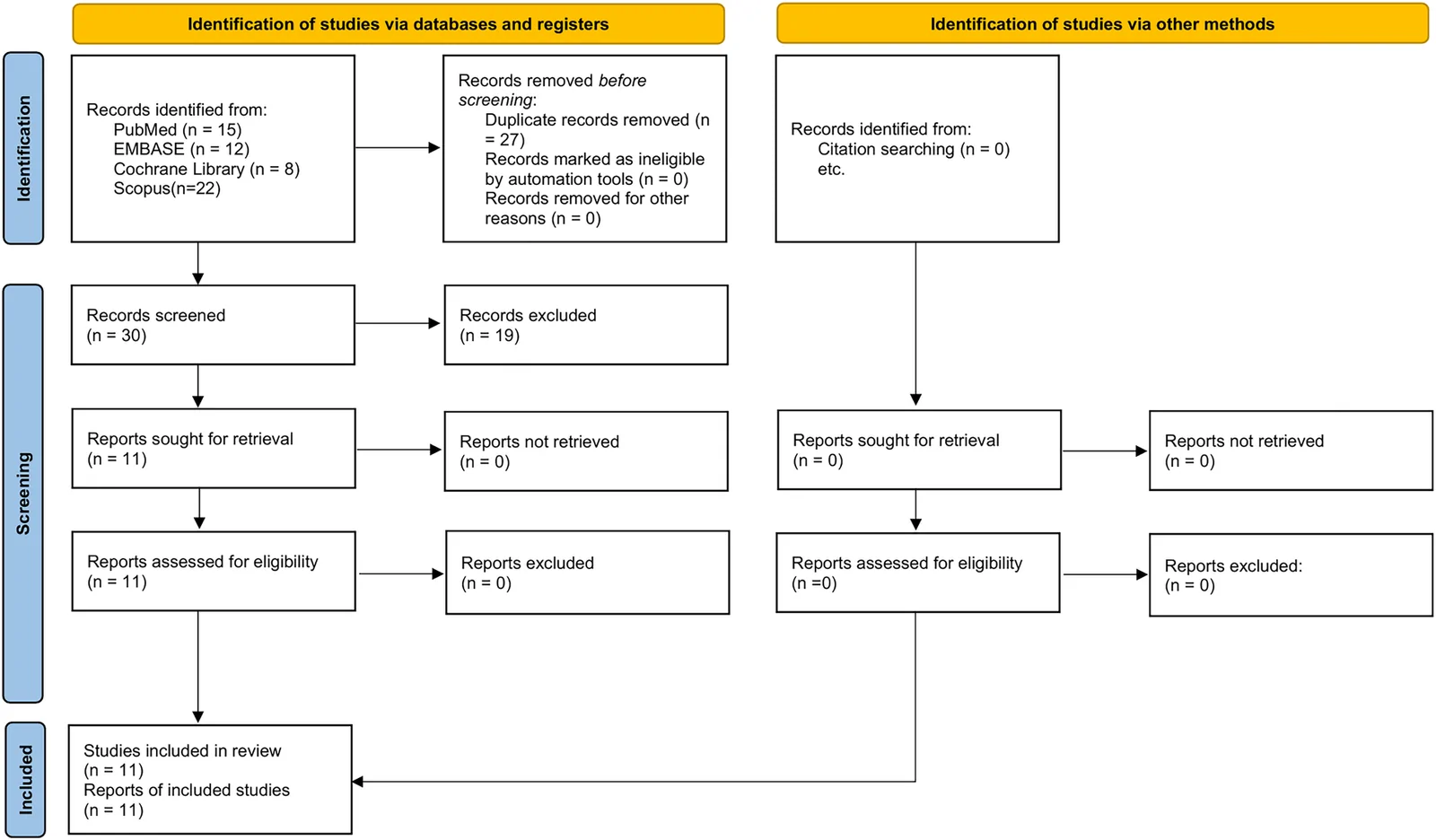
Study selection flow diagram showing the identification, screening, eligibility assessment, and inclusion of studies in the systematic review and meta-analysis.

### Eligibility criteria

Studies were selected according to the Population, Intervention, Comparison, Outcomes, and Study design (PICOS) framework. Eligible populations included patients undergoing THA or TKA. The intervention of interest was intraoperative application of ORC, including conventional ORC materials, fibrillar ORC, ORC powder, or SURGICEL Powder. Eligible comparators included no ORC, placebo, standard care, active topical hemostatic strategies such as TXA or gelatin-thrombin matrix (GTHM), or TXA alone in studies evaluating ORC as an adjunctive intervention. Eligible studies were required to report at least one blood-loss-related or complication-related outcome. Randomized controlled trials (RCTs) and comparative observational studies were included.

Studies were excluded if they were non-comparative reports, case reports, case series without a control group, reviews, meta-analyses, editorials, conference abstracts without sufficient data, duplicate publications, or studies without extractable outcome data.

### Search strategy

A comprehensive literature search was performed in PubMed, Embase, the Cochrane Library, and Scopus from database inception to March 2026. The search strategy combined Medical Subject Headings and free-text terms related to ORC and arthroplasty, including “oxidized regenerated cellulose,” “Surgicel,” “total hip arthroplasty,” and “total knee arthroplasty.” No language restrictions were applied during the initial search. The reference lists of relevant articles were also screened manually to identify additional eligible studies. The full search strategies are provided as an additional file.

### Study selection and data extraction

All retrieved records were imported into reference-management software, and duplicates were removed. Two reviewers independently screened titles and abstracts to identify potentially eligible studies. The full texts of relevant articles were then assessed according to the predefined eligibility criteria. Disagreements were resolved by discussion or consultation with a third reviewer.

Data were extracted independently by two reviewers using a standardized data-extraction form. Extracted information included first author, publication year, country, study design, sample size, arthroplasty type, patient characteristics, ORC formulation, comparator strategy, perioperative blood-management protocol, outcome definitions, follow-up duration, and numerical outcome data. When necessary, corresponding authors were contacted for additional information. Any discrepancies were resolved by consensus.

### Assessment of risk of bias and methodological quality

The methodological quality of RCTs was assessed using the Cochrane risk-of-bias tool (RoB 2), which evaluates bias arising from the randomization process, deviations from intended interventions, missing outcome data, outcome measurement, and selection of reported results (21). Non-randomized comparative studies were assessed using the Newcastle-Ottawa Scale (NOS), which evaluates selection, comparability, and outcome domains (22). NOS scores of 7 or higher were considered to indicate moderate to high methodological quality. All assessments were conducted independently by two reviewers, and disagreements were resolved through discussion.

### Outcome measures

The primary outcomes were total perioperative blood loss, postoperative or hidden blood loss, and hemoglobin drop/loss. Secondary outcomes included intraoperative blood loss, postoperative hematocrit, transfusion rate, thrombotic events, and overall complications. Outcomes reported in fewer than two studies or with substantial heterogeneity in measurement methods were summarized narratively.

### Data synthesis and statistical analysis

Meta-analyses were performed using Review Manager version 5.4 (23). Continuous outcomes were pooled as MDs with 95% CIs because the included studies reported outcomes using the same measurement units. Dichotomous outcomes were pooled as RRs with 95% CIs. Given the anticipated clinical and methodological heterogeneity across studies, including differences in arthroplasty type, study design, ORC formulation, comparator strategy, and perioperative blood-management protocols, random-effects models were used for the primary analyses. Inverse-variance random-effects models were used for continuous outcomes, and Mantel-Haenszel random-effects models were used for dichotomous outcomes.

Statistical heterogeneity was assessed using the Chi-square test and the I^2^ statistic. I^2^ values of approximately 25%, 50%, and 75% were considered to represent low, moderate, and high heterogeneity, respectively (32). Before pooling, clinical comparability was assessed according to comparator type, surgical procedure, outcome definition, and measurement unit. Quantitative synthesis was performed only when at least two studies were considered sufficiently comparable. When substantial heterogeneity was present, pooled estimates were interpreted cautiously and explored using procedure-specific subgroup analyses and leave-one-out sensitivity analyses where possible. If heterogeneity was very high (approximately I^2^ ≥ 75%), the direction of effect was inconsistent, or outcome definitions were clinically incompatible, findings were summarized narratively rather than used to support definitive conclusions. Sensitivity analyses were conducted for the main outcomes using a leave-one-out approach, and analyses restricted to RCTs were performed when sufficient data were available. Publication bias was not formally assessed because fewer than 10 studies were available for each pooled outcome.

## Results

### Literature search and study characteristics

The study-selection process is summarized in Figure 1. A total of 57 records were identified through database searching, including 15 from PubMed, 12 from Embase, 8 from the Cochrane Library, and 22 from Scopus. After removal of 27 duplicates, 30 records underwent title and abstract screening. Nineteen records were excluded, and 11 full-text articles were assessed for eligibility. No article was excluded after full-text review. Ultimately, 11 studies were included in the systematic review and meta-analysis.

The included studies were published between 2018 and 2026 (9–19). Four studies were RCTs, two were prospective non-randomized comparative studies, and five were retrospective comparative cohort studies. Five studies investigated THA, and six investigated TKA, including one study of simultaneous bilateral TKA. The studies were conducted in Japan, China, and South Korea. ORC interventions included conventional ORC, fibrillar ORC, ORC powder, and SURGICEL Powder. Comparator strategies included blank control, placebo, no ORC, topical TXA, GTHM, and topical TXA alone in an adjunct-design study. The most commonly reported outcomes were perioperative blood-loss variables, hemoglobin or hematocrit changes, transfusion, and complications. The characteristics of included studies are summarized in Table 1.

**Table 1:** Characteristics of the included studies.

Author, year	Country	Design	Procedure	Intervention	Comparator	N (group sizes)	Outcomes	Follow-up
Wang 2018 (9)	China	Retrospective cohort	THA	Oxidized regenerated cellulose	No local hemostatic materials	37/32	Total blood loss, hidden blood loss, Hb decline, transfusion, complications	Perioperative period; follow-up to 3 months
Li 2023 (10)	China	Randomized controlled trial	TKA	Oxidized regenerated cellulose	No local hemostatic materials	35/35	Total blood loss, postoperative/hidden blood loss, Hb decline, hematocrit, transfusion, wound complications	Early postoperative period
Wakasa 2024 (15)	Japan	Randomized controlled trial	THA	Oxidized regenerated cellulose	Topical tranexamic acid (TXA)	55/56	Intraoperative blood loss, Hb decline, hematocrit, transfusion, complications	Perioperative period
Wang 2024 (12)	China	Retrospective cohort	TKA	Oxidized regenerated cellulose/SURGICEL powder	Electrocautery	43/42	Total blood loss, Hb decline, transfusion, wound complications	Early postoperative period
Li 2025 (11)	China	Randomized controlled trial	TKA	Oxidized regenerated cellulose	No local hemostatic material/standard control; topical TXA	34/33/34†	Total blood loss, Hb decline, intraoperative blood loss, transfusion	Early postoperative period
Yoon 2025 (13)	South Korea	Retrospective cohort	THA	Oxidized regenerated cellulose powder	Electrocautery	141/138	Total blood loss, postoperative/hidden blood loss, hematocrit, transfusion, complications	Perioperative period; follow-up >3 months
Ishikura 2025 (14)	Japan	Prospective comparative study	THA	Oxidized regenerated cellulose	No ORC/routine management	74/78	Total blood loss, postoperative/hidden blood loss, Hb decline, hematocrit, laboratory indices, thigh swelling	Perioperative period; follow-up to 3 months
Kaneko 2025 (16)	Japan	Retrospective cohort	THA	ORC powder + topical TXA	Topical TXA alone	53/80	Total blood loss, postoperative/hidden blood loss, hematocrit, complications	Perioperative period
Sato 2025 (17)	Japan	Retrospective cohort	TKA (simultaneous bilateral TKA)	Oxidized regenerated cellulose	No ORC/standard care	21/28*	Postoperative/hidden blood loss, Hb/hematocrit change, transfusion-related data	Early postoperative period
Ueyama 2026 (18)	Japan	Randomized controlled trial	TKA	Intraoperative oxidized regenerated cellulose	Standard care/no ORC	50/50	Blood loss, thigh swelling, postoperative pain, complications	Early postoperative period
Hanada 2026 (19)	Japan	Three-arm prospective comparative study	TKA	Oxidized regenerated cellulose	No ORC/standard control; GTHM	10/10/10†	Postoperative/hidden blood loss, Hb decline, intraoperative blood loss	Early postoperative period

ORC, oxidized regenerated cellulose; THA, total hip arthroplasty; TKA, total knee arthroplasty; TXA, tranexamic acid; I/C, intervention/control; GTHM, gelatin–thrombin hemostatic matrix. *The original study involved simultaneous bilateral TKA; sample size should be interpreted according to the unit reported in the source study. †For three-arm studies, sample sizes are shown as ORC/standard-control/active-comparator. Hb, hemoglobin.

### Risk of bias and methodological quality

Four randomized controlled trials were assessed using the Cochrane risk-of-bias tool, and non-randomized comparative studies were evaluated using the Newcastle-Ottawa Scale (21, 22). In the randomized trials, blinding of participants and personnel was generally judged to be at high risk because the interventions were intraoperative topical procedures. Outcome assessment, incomplete outcome data, and other bias were judged to be at low risk in most or all randomized trials, whereas random sequence generation, allocation concealment, and selective reporting were unclear in some studies. The risk-of-bias graph and risk-of-bias summary for randomized controlled trials are shown in Figures 2, 3, respectively.

**Figure 2:**
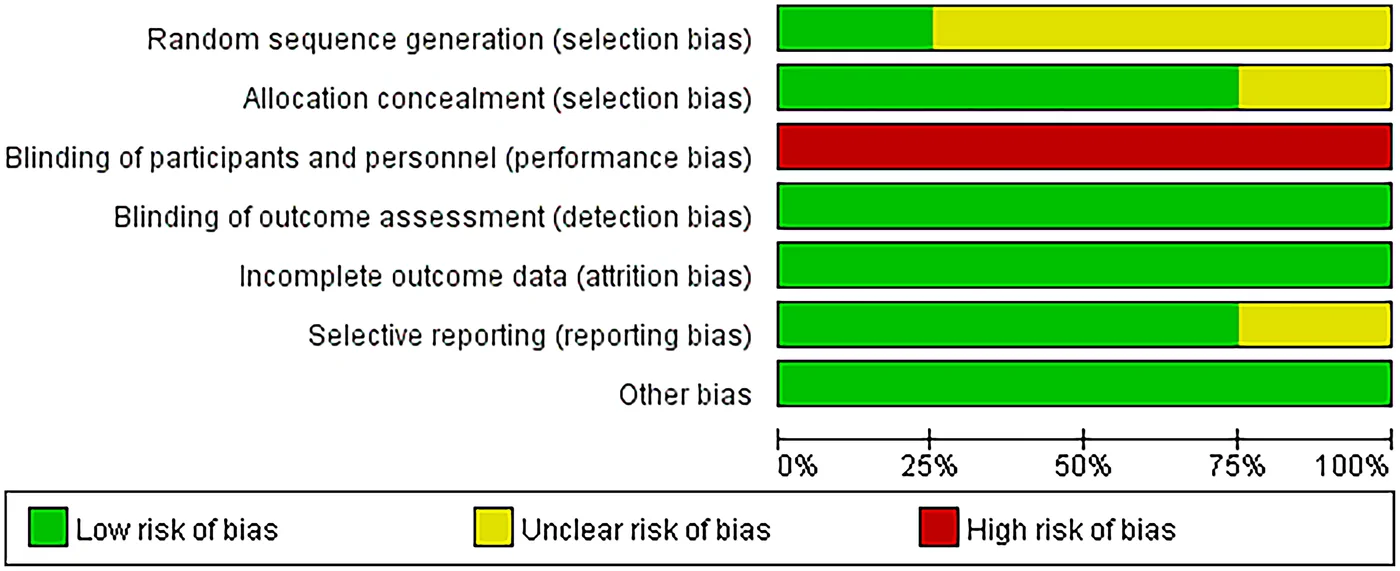
Risk-of-bias graph for randomized controlled trials included in the systematic review.

**Figure 3:**
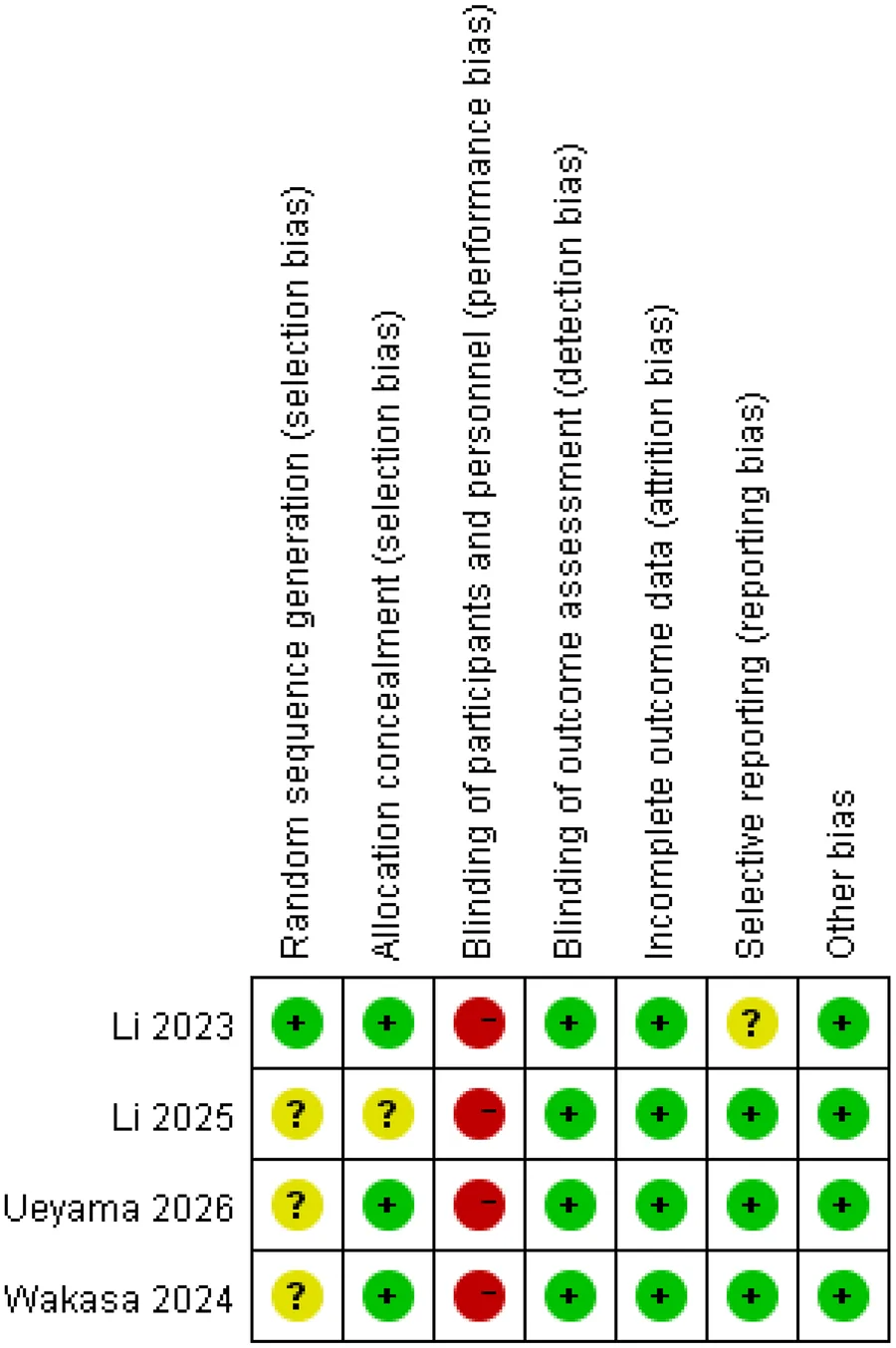
Risk-of-bias summary for randomized controlled trials included in the systematic review.

For the non-randomized comparative studies with available NOS assessments, total scores ranged from 7 to 8 points, indicating moderate to high methodological quality. The main reasons for score reduction were limited adjustment for additional confounders and incomplete or inconsistent reporting of follow-up completeness or outcome assessment methods in some cohorts. Detailed NOS assessments are summarized in Table 2.

**Table 2:** Quality assessment of non-randomized studies using the Newcastle-Ottawa scale.

Study	Study design	Selection (max 4)	Comparability (max 2)	Outcome (max 3)	Total score	Quality level
Wang 2018 (9)	Retrospective cohort	4	2	2	8/9	High quality
Yoon 2025 (13)	Retrospective cohort	4	2	1	7/9	Moderate quality
Ishikura 2025 (14)	Prospective interventional comparative study	4	2	2	8/9	High quality
Kaneko 2025 (16)	Retrospective cohort	4	2	1	7/9	Moderate quality
Sato 2025 (17)	Retrospective cohort	4	2	1	7/9	Moderate quality
Wang 2024 (12)	Retrospective cohort	4	2	1	7/9	Moderate quality

NOS, newcastle-ottawa scale. The NOS evaluates methodological quality based on three domains: selection (0–4 points), comparability (0–2 points), and outcome (0–3 points), with a maximum score of 9 points. Studies with total scores of 7–9 points were considered moderate to high quality.

### Quantitative synthesis

#### ORC vs. no ORC or standard control

Compared with no ORC or standard control, ORC was associated with lower total perioperative blood loss (MD = −124.77 mL, 95% CI −181.71 to −67.83; *P* < 0.0001; I^2^ = 48%). Reductions were observed in both the TKA and THA subgroups. ORC was also associated with lower postoperative or hidden blood loss (MD = −136.54 mL, 95% CI −214.97 to −58.10; *P* = 0.0006; I^2^ = 74%) and hemoglobin drop/loss (MD = −0.57 g/dL, 95% CI −0.81 to −0.34; *P* < 0.00001; I^2^ = 33%). No significant between-group differences were found for intraoperative blood loss, transfusion rate, thrombotic events, or overall complications. In the single study reporting postoperative hematocrit, postoperative hematocrit was higher in the ORC group (MD = 1.00%, 95% CI 0.21–1.79; *P* = 0.01). Because the main statistically significant findings were based on blood-loss-related surrogate outcomes and were not consistently accompanied by significant improvements in transfusion or complication outcomes, their clinical importance should be interpreted cautiously. The forest plots for the primary blood-loss-related outcomes are shown in Figure 4, and the forest plots for safety and clinically relevant secondary outcomes are shown in Figure 5.

**Figure 4:**
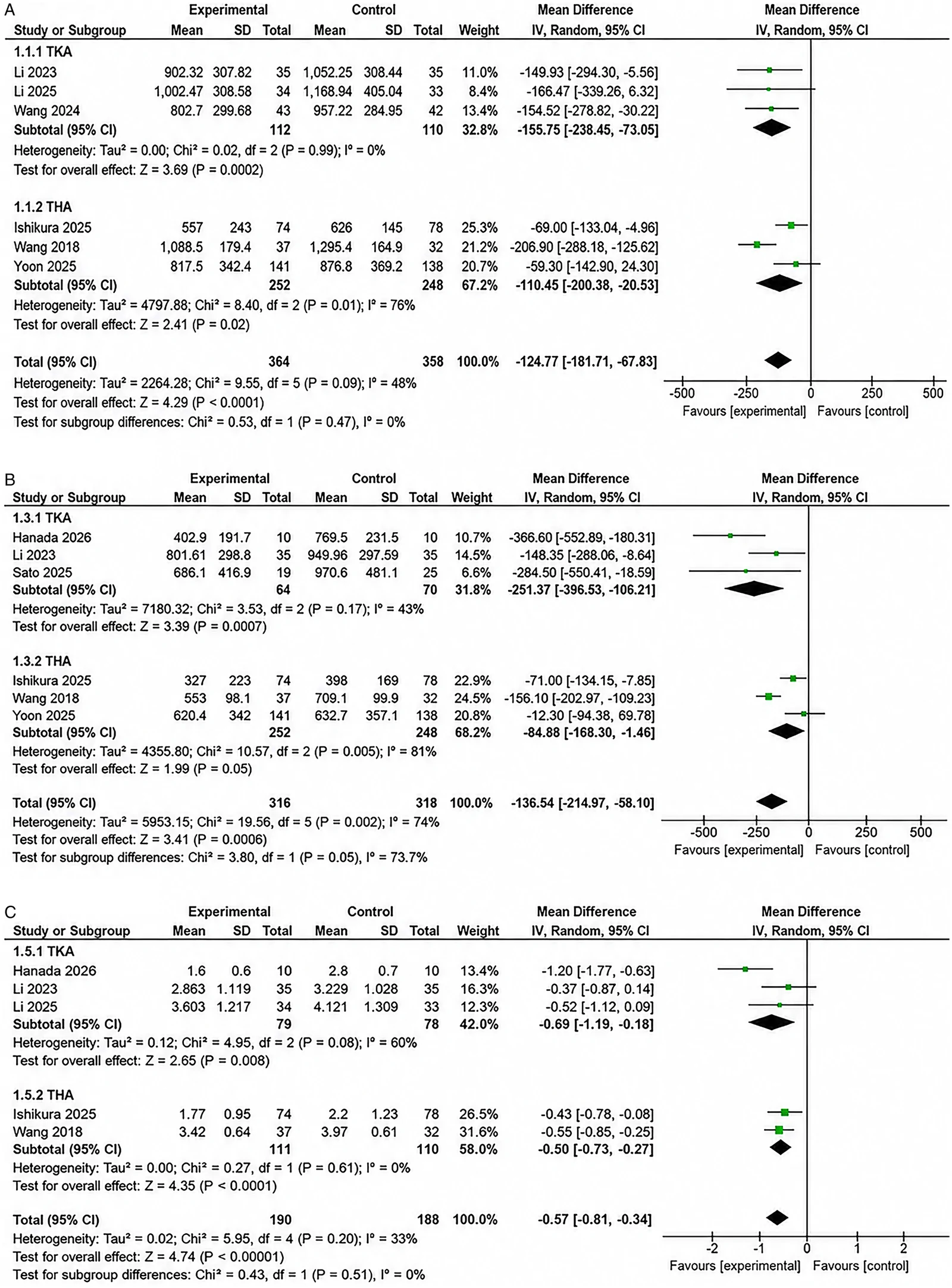
Forest plots comparing ORC with no ORC or standard control for primary blood-loss-related outcomes: **(A)** total perioperative blood loss, **(B)** postoperative or hidden blood loss, and **(C)** hemoglobin drop/loss. ORC, oxidized regenerated cellulose; CI, confidence interval.

**Figure 5:**
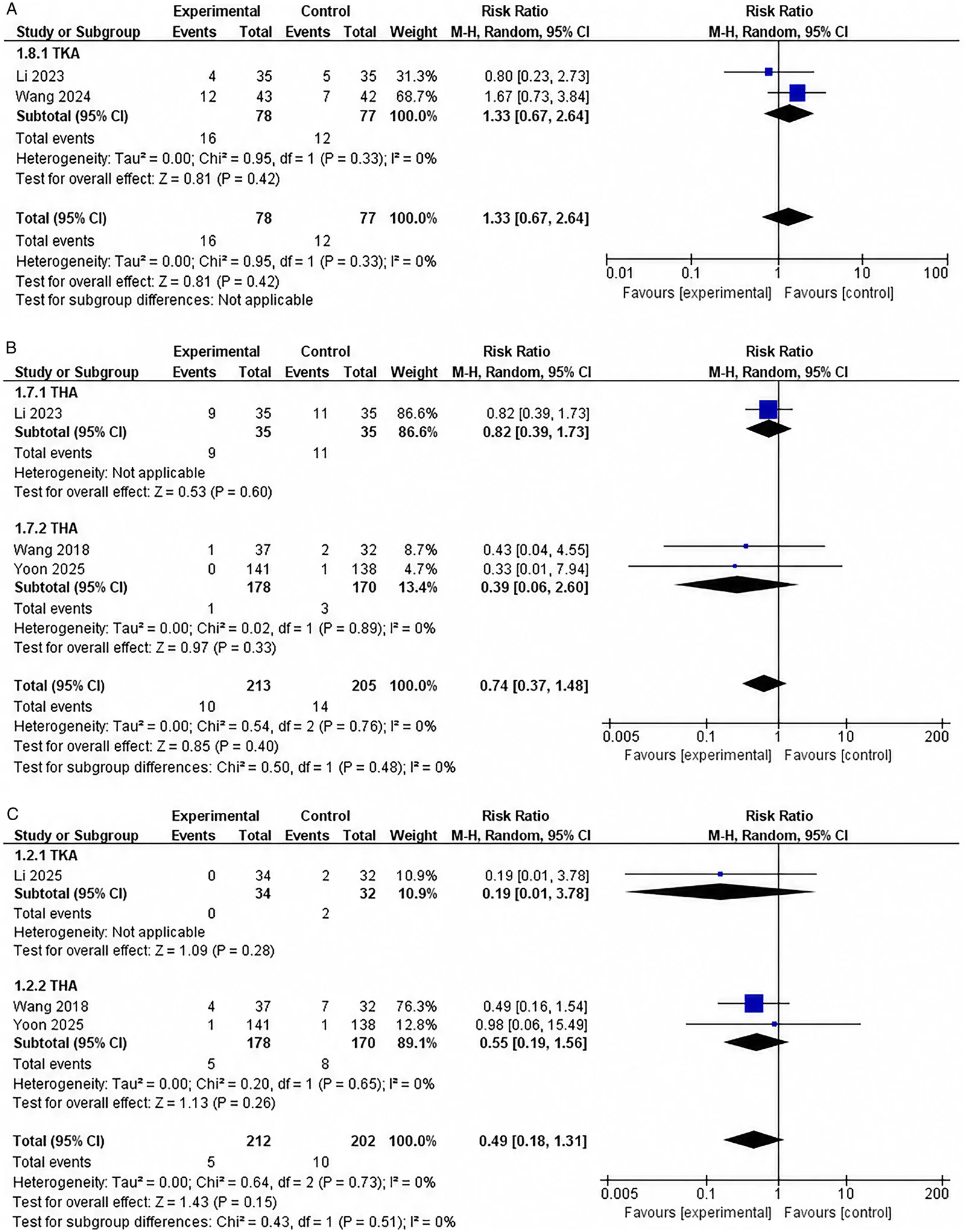
Forest plots comparing ORC with no ORC or standard control for safety and clinically relevant secondary outcomes: **(A)** overall complications, **(B)** thrombotic events, and **(C)** transfusion rate. ORC, oxidized regenerated cellulose; CI, confidence interval.

#### ORC vs. active topical comparator

When ORC was compared with active topical comparators such as topical TXA or GTHM, no significant differences were found in total perioperative blood loss (MD = 57.86 mL, 95% CI −34.75–150.47; *P* = 0.22; I^2^ = 0%), postoperative or hidden blood loss (MD = 38.72 mL, 95% CI −106.77–184.22; *P* = 0.60; I^2^ = 53%), hemoglobin drop/loss (MD = −0.12 g/dL, 95% CI −0.68–0.44; *P* = 0.68; I^2^ = 55%), or postoperative hematocrit (MD = −0.10%, 95% CI −1.46–1.26; *P* = 0.89). Transfusion rate was not estimable because no events occurred in either group. The forest plots for this comparison are shown in Figure 6.

**Figure 6:**
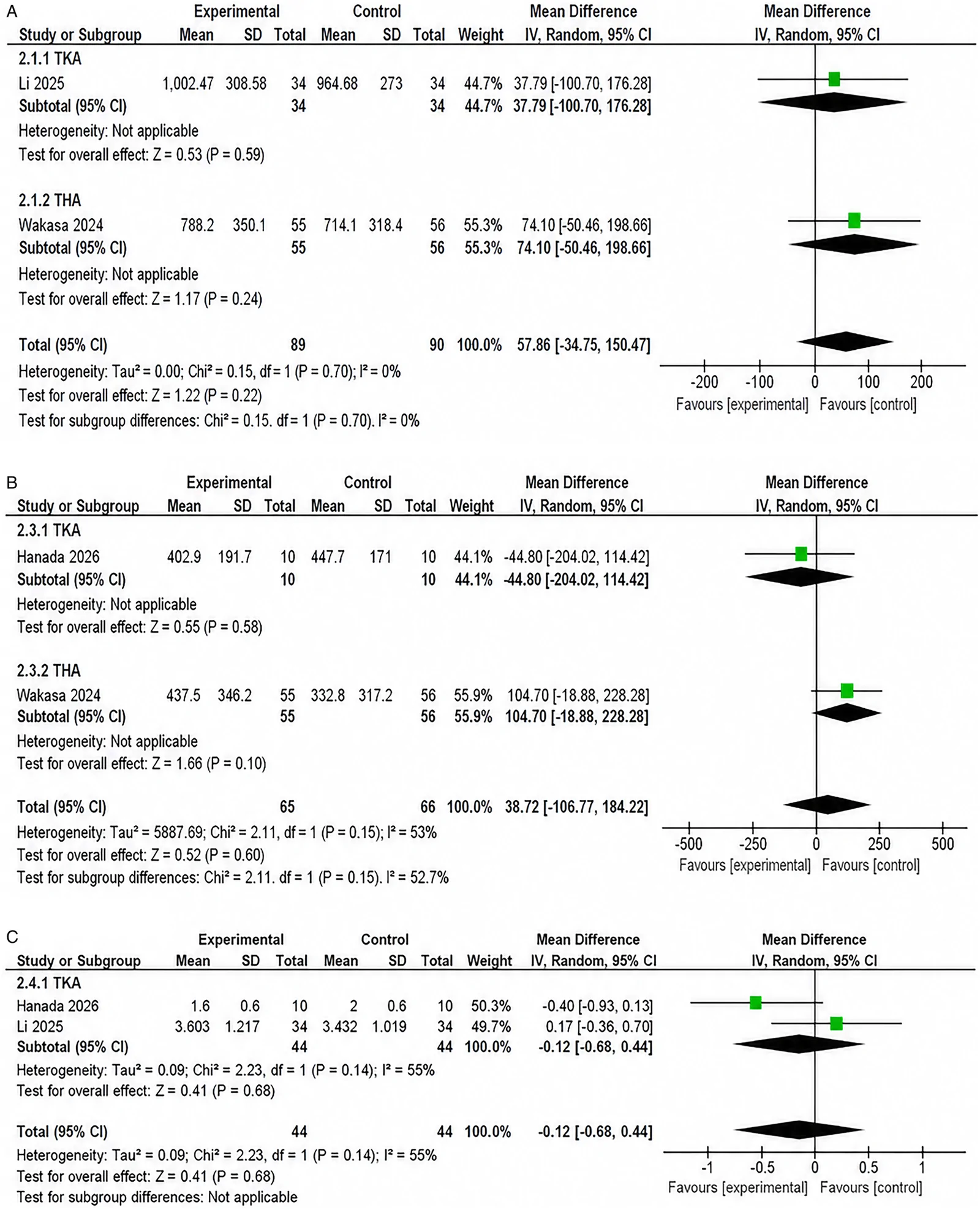
Forest plots comparing ORC with active topical comparators for blood-loss-related outcomes: **(A)** total perioperative blood loss, **(B)** postoperative or hidden blood loss, and **(C)** hemoglobin drop/loss. ORC, oxidized regenerated cellulose; CI, confidence interval.

#### ORC plus TXA vs. TXA alone

In the single available study comparing ORC plus TXA with TXA alone, ORC plus TXA reduced total perioperative blood loss (MD = −196.70 mL, 95% CI −285.90 to −107.50; *P* < 0.0001) and postoperative or hidden blood loss (MD = −134.50 mL, 95% CI −212.56 to −56.44; *P* = 0.0007). Intraoperative blood loss showed a trend toward reduction, but the difference did not reach clear statistical significance (MD = −62.20 mL, 95% CI −125.19–0.79; *P* = 0.05). Overall complications were not estimable because no events occurred in either group. Because this comparison was based on only one study, it was considered exploratory and was not interpreted as definitive evidence of incremental benefit beyond TXA.

#### Sensitivity analyses

Sensitivity analyses were performed for the three core outcomes in the comparison of ORC vs. no ORC or standard control. For total perioperative blood loss and hemoglobin drop/loss, omitting any single study did not materially change the direction or statistical significance of the pooled effects, indicating good robustness. For total perioperative blood loss, exclusion of Wang 2018 substantially reduced heterogeneity, suggesting that this study may have contributed to between-study variability. RCT-only analyses for both outcomes remained statistically significant.

For postoperative or hidden blood loss, the pooled effect consistently favored ORC across leave-one-out analyses; however, substantial heterogeneity persisted, indicating that the observed variability was likely multifactorial rather than driven by a single study. The RCT-only analysis for this outcome was limited because only one randomized study was available.

#### Narrative synthesis of non-pooled outcomes

Several outcomes were too sparse or too heterogeneous for formal pooling, including postoperative thigh swelling, imaging-based swelling parameters, pain scores, inflammatory and coagulation-related laboratory markers, and short-term functional outcomes. Two studies suggested that ORC may reduce postoperative thigh swelling, although the measurement methods differed considerably. In contrast, pain, early functional recovery, and most secondary laboratory indices showed no consistent between-group differences. Overall, these findings suggest that the main measurable benefit of ORC is concentrated in blood-loss-related outcomes, whereas its influence on broader early recovery endpoints remains uncertain.

### Publication bias

Publication bias was not formally assessed because fewer than 10 studies were available for each pooled outcome.

## Discussion

In this systematic review and meta-analysis, ORC was associated with lower total perioperative blood loss, postoperative or hidden blood loss, and hemoglobin decline compared with no ORC or standard control. However, these findings mainly reflected calculated blood-loss-related surrogate outcomes and were not consistently accompanied by reductions in transfusion, thrombotic events, overall complications, or recovery-related outcomes. ORC did not show clear superiority over active topical hemostatic strategies, and evidence for its use as an adjunct to TXA remained limited and exploratory (24, 25).

Although the pooled reductions were statistically significant in the standard-control comparison, their clinical importance remains uncertain. A universally accepted minimal clinically important difference for perioperative blood loss in THA or TKA has not been established. Because improvements in transfusion, pain, functional recovery, length of stay, or safety outcomes were not consistently demonstrated, the observed effects should be interpreted mainly as improvements in surrogate outcomes rather than definitive patient-important benefits (9–19).

The observed pattern is biologically plausible. ORC may be particularly relevant for diffuse oozing from osteotomy surfaces, exposed cancellous bone, synovectomy or soft-tissue release in TKA, and acetabular, femoral medullary, or periarticular bleeding in THA (9, 10, 12–14, 17, 18, 27–29). This anatomical rationale may explain why ORC appeared more consistently related to postoperative or hidden blood-loss outcomes than to immediately measured intraoperative blood loss.

The comparator-specific findings are central to interpretation. Standard-control studies generally favored ORC for blood-loss-related outcomes, whereas trials or comparative studies against topical TXA or GTHM showed broadly comparable effects rather than clear ORC superiority (9–19). Thus, the current evidence supports ORC as a possible local hemostatic option, but not as an established superior alternative to active topical agents (30, 31).

The role of ORC in TXA-based protocols also remains uncertain. Limited evidence suggests potential additional blood-loss reduction when ORC is combined with TXA, which is mechanistically plausible because TXA inhibits fibrinolysis whereas ORC provides a local matrix for clot formation (16). However, the available evidence is insufficient to establish routine incremental benefit.

Recovery-related outcomes provide an important counterbalance to the blood-loss findings. Although some studies reported less swelling or pain after ORC application, broader recovery outcomes such as functional scores, mobility, and short-term clinical recovery were not consistently improved (14–16, 18). This pattern reinforces that the current evidence primarily supports a hemostatic signal, while broader recovery benefits remain uncertain.

The main added value of this review is its comparator-stratified framework. Compared with a previous meta-analysis that mainly summarized ORC against heterogeneous control strategies, the present review separates no-local-hemostatic control, active topical comparator, and TXA-adjunct designs (26). This distinction matters because these comparisons answer different clinical questions, especially in the current TXA era, where the key issue is whether ORC adds clinically meaningful value beyond established blood-management strategies.

The review also has limitations. The included studies varied in design, procedure, surgical approach, ORC formulation and dose, application site, comparator treatment, TXA use, tourniquet and drainage protocols, anticoagulation, transfusion criteria, measurement time points, and blood-loss calculation methods. These factors are credible sources of clinical and methodological heterogeneity, particularly for postoperative or hidden blood loss. Therefore, pooled estimates with substantial heterogeneity should be interpreted cautiously and not as evidence of a uniform treatment effect. Several outcomes, including transfusion, thrombotic events, overall complications, and recovery-related endpoints, were limited by low event numbers or inconsistent reporting. Some comparisons, especially ORC as an adjunct to TXA, were based on limited evidence, and publication-bias assessment was not possible because fewer than 10 studies were available for each pooled outcome (32, 33).

## Conclusions

Current evidence suggests that ORC may modestly reduce blood-loss-related surrogate outcomes in THA and TKA, especially compared with no ORC or standard care. However, consistent benefits for transfusion, recovery, or safety outcomes have not been established. ORC has not shown clear superiority over active topical hemostatic comparators, and its incremental value in TXA-based protocols remains limited and exploratory. Because the overall certainty of evidence remains limited, routine ORC use in unselected arthroplasty patients therefore cannot be recommended based on current evidence. Further well-designed trials should determine whether ORC offers clinically meaningful and cost-effective benefit in selected patients or procedures with high bleeding risk or difficult cancellous-bone or diffuse soft-tissue oozing.

## Data Availability

The original contributions presented in the study are included in the article/Supplementary Material, further inquiries can be directed to the corresponding author.
