# Supplementary material for: Topical oxidized regenerated cellulose for perioperative blood management in total hip and knee arthroplasty: a systematic review and meta-analysis
Source: Front Surg. 2026 Sep 18;13:1900156. doi: 10.3389/fsurg.2026.1900156 (PMC13630781; doi:10.3389/fsurg.2026.1900156)
Supplement: Supplementary file 2 [file Table2.docx]

**Supplementary Material 1. Complete Search Strategies**

**Supplementary Material 1.** Complete database-specific search strategies used for PubMed, Embase, the Cochrane Library, and Scopus. Searches were performed from database inception to March 2026. No language restrictions were applied during the initial search. The number of records retrieved from each database is shown before duplicate removal.

| **Database** | **Platform / interface** | **Date last searched** | **Records retrieved** |
| --- | --- | --- | --- |
| PubMed | PubMed | March 2026 | 15 |
| Embase | Embase.com | March 2026 | 12 |
| Cochrane Library | Cochrane Library | March 2026 | 8 |
| Scopus | Scopus Advanced Search | March 2026 | 22 |

**PubMed**

**Search date:** March 2026

**Limits / filters:** No language, date, article-type, or publication-status restrictions were applied.

**Records retrieved:** n = 15

**Search strategy:**

(

"Cellulose, Oxidized"[Mesh]

OR "oxidized regenerated cellulose"[Title/Abstract]

OR "oxidised regenerated cellulose"[Title/Abstract]

OR "oxidized cellulose"[Title/Abstract]

OR "oxidised cellulose"[Title/Abstract]

OR Surgicel[Title/Abstract]

OR "SURGICEL Powder"[Title/Abstract]

)

AND

(

"Arthroplasty, Replacement, Hip"[Mesh]

OR "Arthroplasty, Replacement, Knee"[Mesh]

OR "total hip arthroplasty"[Title/Abstract]

OR "total knee arthroplasty"[Title/Abstract]

OR "hip arthroplasty"[Title/Abstract]

OR "knee arthroplasty"[Title/Abstract]

OR "total hip replacement"[Title/Abstract]

OR "total knee replacement"[Title/Abstract]

OR "hip replacement"[Title/Abstract]

OR "knee replacement"[Title/Abstract]

)

**Embase**

**Search date:** March 2026

**Interface:** Embase.com

**Limits / filters:** No language, date, article-type, or publication-status restrictions were applied.

**Records retrieved:** n = 12

**Search strategy:**

(

'oxidized cellulose'/exp

OR 'oxidized regenerated cellulose':ti,ab,kw

OR 'oxidised regenerated cellulose':ti,ab,kw

OR 'oxidized cellulose':ti,ab,kw

OR 'oxidised cellulose':ti,ab,kw

OR surgicel:ti,ab,kw

OR 'surgicel powder':ti,ab,kw

)

AND

(

'total hip arthroplasty'/exp

OR 'total knee arthroplasty'/exp

OR 'hip arthroplasty'/exp

OR 'knee arthroplasty'/exp

OR 'total hip arthroplasty':ti,ab,kw

OR 'total knee arthroplasty':ti,ab,kw

OR 'hip arthroplasty':ti,ab,kw

OR 'knee arthroplasty':ti,ab,kw

OR 'total hip replacement':ti,ab,kw

OR 'total knee replacement':ti,ab,kw

OR 'hip replacement':ti,ab,kw

OR 'knee replacement':ti,ab,kw

)

**Cochrane Library**

**Search date:** March 2026

**Limits / filters:** No language, date, article-type, or publication-status restrictions were applied.

**Records retrieved:** n = 8

**Search strategy:**

(

"oxidized regenerated cellulose"

OR "oxidised regenerated cellulose"

OR "oxidized cellulose"

OR "oxidised cellulose"

OR Surgicel

OR "SURGICEL Powder"

)

AND

(

"total hip arthroplasty"

OR "total knee arthroplasty"

OR "hip arthroplasty"

OR "knee arthroplasty"

OR "total hip replacement"

OR "total knee replacement"

OR "hip replacement"

OR "knee replacement"

)

**Scopus**

**Search date:** March 2026

**Interface:** Scopus Advanced Search

**Limits / filters:** No language, date, document-type, or publication-status restrictions were applied.

**Records retrieved:** n = 22

**Search strategy:**

TITLE-ABS-KEY(

"oxidized regenerated cellulose"

OR "oxidised regenerated cellulose"

OR "oxidized cellulose"

OR "oxidised cellulose"

OR surgicel

OR "surgicel powder"

)

AND

TITLE-ABS-KEY(

"total hip arthroplasty"

OR "total knee arthroplasty"

OR "hip arthroplasty"

OR "knee arthroplasty"

OR "total hip replacement"

OR "total knee replacement"

OR "hip replacement"

OR "knee replacement"

)

**Manual Reference-List Screening**

**Source searched:** Reference lists of relevant articles and previous reviews.

**Search date:** March 2026

**Additional eligible records identified:** n = 0
