# Supplementary material for: Topical oxidized regenerated cellulose for perioperative blood management in total hip and knee arthroplasty: a systematic review and meta-analysis
Source: Front Surg. 2026 Sep 18;13:1900156. doi: 10.3389/fsurg.2026.1900156 (PMC13630781; doi:10.3389/fsurg.2026.1900156)
Supplement: Supplementary file 3 [file Table3.docx]

Supplementary Material 3. GRADE Summary of Findings

**Caption.** Certainty of evidence for key outcomes in the systematic review and meta-analysis of oxidized regenerated cellulose in total hip and knee arthroplasty. Certainty was assessed according to GRADE domains: risk of bias, inconsistency, indirectness, imprecision, and publication bias. Certainty ratings should be interpreted together with the comparator category because standard-care controls, active topical comparators, and adjunctive tranexamic acid designs address different clinical questions.

**Abbreviations:** CI, confidence interval; GRADE, Grading of Recommendations Assessment, Development and Evaluation; GTHM, gelatin-thrombin hemostatic matrix; MD, mean difference; ORC, oxidized regenerated cellulose; RR, risk ratio; TXA, tranexamic acid.

Table S3.1. ORC versus no ORC or standard care

| **Outcome** | **Studies and participants** | **Effect estimate** | **Certainty** | **Reasons for rating** | **Interpretation** |
| --- | --- | --- | --- | --- | --- |
| Total perioperative blood loss | Comparative studies included in the primary analysis; mixed randomized and non-randomized evidence | MD -124.77 mL (95% CI -181.71 to -67.83); I2 = 48% | Low | Downgraded for risk of bias from mixed study designs and some methodological limitations; inconsistency was moderate. | ORC may modestly reduce calculated total perioperative blood loss compared with no ORC or standard care. |
| Postoperative or hidden blood loss | Comparative studies included in the primary analysis; outcome definitions varied across studies | MD -136.54 mL (95% CI -214.97 to -58.10); I2 = 74% | Low | Downgraded for risk of bias and substantial inconsistency related to outcome definitions, calculation methods, and perioperative protocols. | ORC may reduce postoperative or hidden blood loss, but the magnitude of effect is uncertain because heterogeneity was substantial. |
| Hemoglobin drop or loss | Comparative studies included in the primary analysis | MD -0.57 g/dL (95% CI -0.81 to -0.34); I2 = 33% | Low | Downgraded for risk of bias and mixed study designs; inconsistency was not substantial. | ORC may modestly reduce hemoglobin decline compared with no ORC or standard care. |
| Intraoperative blood loss | Limited studies reported this outcome in the standard-care comparison | No statistically significant difference reported in the pooled analysis | Very low | Downgraded for risk of bias, indirectness because ORC is usually applied near the end of surgery, and imprecision. | Current evidence does not show a reliable intraoperative blood-loss benefit. |
| Transfusion rate | Three studies; 414 participants in the pooled analysis | RR 0.49 (95% CI 0.18 to 1.31); I2 = 0% | Very low | Downgraded for sparse events and imprecision; confidence interval is compatible with benefit or no important effect. | Evidence is insufficient to determine whether ORC reduces transfusion requirements. |
| Thrombotic events | Three studies; 418 participants in the pooled analysis | RR 0.74 (95% CI 0.37 to 1.48); I2 = 0% | Very low | Downgraded for sparse events, imprecision, and limited systematic screening for asymptomatic thromboembolism. | No clear safety signal was detected, but rare or asymptomatic thrombotic events cannot be excluded. |
| Overall complications | Two studies; 155 participants in the pooled analysis | RR 1.33 (95% CI 0.67 to 2.64); I2 = 0% | Very low | Downgraded for sparse events, imprecision, and heterogeneous definitions of overall complications. | Evidence is insufficient to determine whether ORC affects overall complication risk. |

Table S3.2. ORC versus active topical hemostatic comparators

| **Outcome** | **Studies and participants** | **Effect estimate** | **Certainty** | **Reasons for rating** | **Interpretation** |
| --- | --- | --- | --- | --- | --- |
| Total perioperative blood loss | Studies comparing ORC with active topical agents such as topical TXA or GTHM | MD 57.86 mL (95% CI -34.75 to 150.47); I2 = 0% | Very low | Downgraded for small number of studies, imprecision, and risk of bias; studies were not designed as equivalence or non-inferiority trials. | Available evidence does not demonstrate a clear difference between ORC and active topical comparators. This should not be interpreted as equivalence. |
| Postoperative or hidden blood loss | Studies comparing ORC with active topical agents | MD 38.72 mL (95% CI -106.77 to 184.22); I2 = 53% | Very low | Downgraded for risk of bias, moderate inconsistency, and wide confidence interval. | The comparative effect of ORC versus active topical agents on postoperative or hidden blood loss remains uncertain. |
| Hemoglobin drop or loss | Studies comparing ORC with active topical agents | MD -0.12 g/dL (95% CI -0.68 to 0.44); I2 = 55% | Very low | Downgraded for risk of bias, moderate inconsistency, and imprecision. | No clear difference was demonstrated, but the evidence is too uncertain to support equivalence. |
| Postoperative hematocrit | Studies comparing ORC with active topical agents | MD -0.10% (95% CI -1.46 to 1.26) | Very low | Downgraded for limited data and imprecision. | Evidence is insufficient to determine whether ORC differs from active comparators for postoperative hematocrit. |
| Transfusion rate | Studies comparing ORC with active topical agents | Not estimable because no transfusion events occurred in the available studies | Very low | Downgraded for very sparse events and inability to estimate a pooled effect. | No conclusion can be drawn about comparative transfusion risk. |

Table S3.3. ORC plus TXA versus TXA alone

| **Outcome** | **Studies and participants** | **Effect estimate** | **Certainty** | **Reasons for rating** | **Interpretation** |
| --- | --- | --- | --- | --- | --- |
| Total perioperative blood loss | One study comparing ORC plus TXA with TXA alone | MD -196.70 mL (95% CI -285.90 to -107.50) | Very low | Downgraded for single-study evidence, risk of bias, and indirectness because findings may depend on the specific TXA protocol and ORC application method. | ORC plus TXA may reduce calculated total blood loss compared with TXA alone, but the finding is exploratory. |
| Postoperative or hidden blood loss | One study comparing ORC plus TXA with TXA alone | MD -134.50 mL (95% CI -212.56 to -56.44) | Very low | Downgraded for single-study evidence, risk of bias, and indirectness. | ORC plus TXA may reduce postoperative or hidden blood loss, but the certainty is very low. |
| Intraoperative blood loss | One study comparing ORC plus TXA with TXA alone | MD -62.20 mL (95% CI -125.19 to 0.79) | Very low | Downgraded for single-study evidence and imprecision; the confidence interval includes no clear effect. | The effect on intraoperative blood loss remains uncertain. |
| Safety outcomes | One study comparing ORC plus TXA with TXA alone | Not estimable or inadequately powered because events were absent or sparse | Very low | Downgraded for sparse events and single-study evidence. | The safety of adding ORC to TXA cannot be established from the available evidence. |

GRADE interpretation

High certainty: further research is very unlikely to change confidence in the estimate of effect.

Moderate certainty: further research may change confidence in the estimate of effect.

Low certainty: further research is likely to have an important impact on confidence in the estimate of effect and may change the estimate.

Very low certainty: the true effect is likely to be substantially different from the estimate of effect.

For outcomes based on mixed randomized and non-randomized evidence, certainty was judged conservatively according to study design limitations, risk of bias, inconsistency, imprecision, and clinical indirectness.
