# Supplementary material for: Topical oxidized regenerated cellulose for perioperative blood management in total hip and knee arthroplasty: a systematic review and meta-analysis
Source: Front Surg. 2026 Sep 18;13:1900156. doi: 10.3389/fsurg.2026.1900156 (PMC13630781; doi:10.3389/fsurg.2026.1900156)
Supplement: Supplementary file 4 [file Table4.docx]

**Supplementary Material 4. Risk-of-bias and methodological quality assessment**

Caption. Detailed risk-of-bias assessment for randomized controlled trials using the Cochrane Risk of Bias 2 tool and methodological quality assessment for non-randomized comparative studies using the Newcastle-Ottawa Scale. Judgments were based on the methods and outcome reporting described in the included studies.

Abbreviations. ORC, oxidized regenerated cellulose; RCT, randomized controlled trial; RoB 2, Cochrane Risk of Bias 2 tool; NOS, Newcastle-Ottawa Scale; GTHM, gelatin-thrombin hemostatic matrix; TXA, tranexamic acid.

**Table S4.1. Risk-of-bias assessment for randomized controlled trials using RoB 2.**

| **Study** | **Randomization process** | **Deviations from intended interventions** | **Missing outcome data** | **Measurement of outcome** | **Selection of reported result** | **Overall judgment** | **Main reason for overall judgment** |
| --- | --- | --- | --- | --- | --- | --- | --- |
| Li 2023 | Some concerns | Some concerns | Low risk | Some concerns | Some concerns | Some concerns | Randomization was reported, but allocation concealment and protocol-based selective reporting were not fully described; blinding of intraoperative treatment was difficult. |
| Wakasa 2024 | Low risk | Some concerns | Low risk | Low risk | Some concerns | Some concerns | Randomized design and outcome reporting were generally clear, but the intraoperative nature of the intervention limited blinding and no formal equivalence design was used. |
| Li 2025 | Some concerns | Some concerns | Low risk | Some concerns | Some concerns | Some concerns | The trial was prospective and randomized, but allocation concealment, blinding, and prospective reporting of all prespecified outcomes were not fully detailed. |
| Ueyama 2026 | Low risk | Some concerns | Low risk | Low risk | Some concerns | Some concerns | The study was reported as a single-blind RCT, but surgeon blinding was not feasible and protocol registration or selective reporting details were limited. |

Note. The overall judgment followed the RoB 2 principle that the overall risk of bias reflects the highest level of concern across domains. No randomized trial was judged at low overall risk because at least one domain raised some concerns.

**Table S4.2. Methodological quality assessment of non-randomized comparative studies using the Newcastle-Ottawa Scale.**

| **Study** | **Study design** | **Selection (0-4)** | **Comparability (0-2)** | **Outcome (0-3)** | **Total score** | **Quality level** | **Main reason for score reduction** |
| --- | --- | --- | --- | --- | --- | --- | --- |
| Wang 2018 | Retrospective comparative cohort | 4 | 2 | 2 | 8/9 | High | Follow-up and outcome assessment were not described in enough detail for the maximum outcome score. |
| Wang 2024 | Retrospective comparative cohort | 4 | 2 | 1 | 7/9 | Moderate | Retrospective design and limited follow-up or outcome-assessment details reduced the outcome domain score. |
| Yoon 2025 | Retrospective comparative cohort | 4 | 2 | 1 | 7/9 | Moderate | Outcome assessment and follow-up completeness were not fully detailed. |
| Ishikura 2025 | Prospective non-randomized comparative study | 4 | 2 | 2 | 8/9 | High | Non-randomized allocation remained a methodological limitation despite prospective data collection. |
| Kaneko 2025 | Retrospective comparative cohort | 4 | 2 | 1 | 7/9 | Moderate | Retrospective design and limited outcome-assessment details reduced confidence. |
| Sato 2025 | Retrospective comparative cohort | 4 | 2 | 1 | 7/9 | Moderate | Retrospective design and limited reporting of follow-up completeness reduced the outcome score. |
| Hanada 2026 | Prospective comparative study | 4 | 1 | 2 | 7/9 | Moderate | Small group sizes and limited adjustment for confounders reduced the comparability score. |

Note. NOS scores of 7 to 9 points were considered to indicate moderate to high methodological quality. The score summarizes study-level methodological quality and does not remove the possibility of residual confounding in non-randomized studies.

**Overall interpretation**

The randomized trials generally provided direct comparative evidence, but their overall risk of bias was judged as some concerns because blinding of intraoperative topical interventions was difficult and selective-reporting information was limited. The non-randomized studies were rated as moderate to high methodological quality by the NOS, but residual confounding, retrospective design in several cohorts, and incomplete outcome-assessment details should be considered when interpreting pooled estimates.
